# Evaluating the Cognitive Effects of Video-Induced Negative Affect in College Students: A Comparative Study between Acute Exercise and Music Listening

**DOI:** 10.3390/jintelligence11010012

**Published:** 2023-01-05

**Authors:** Chaoxin Ji, Jun Yang, Lin Lin, Song Chen

**Affiliations:** 1 Physical Education Department, Northeastern University, Shenyang 110819, China; 2 College of Information Science and Engineering, Northeastern University, Shenyang 110819, China; 3 School of Social and Political Science, University of Glasgow, Glasgow G12 8QQ, UK

**Keywords:** cognition, video-induced negative affect, acute exercise, music listening, college students

## Abstract

Background: Video-induced negative affect may have an impact on cognition. In this study, acute exercise and music listening are used to explore their impact on individual cognition with video-induced negative affect. Method: All the participants were randomly divided into six groups. Group 1 (*n* = 19, average age = 20.15) was not given any form of acute exercise or music listening; Group 2 (*n* = 20, average age = 21.33) was given music listening; Group 3 (*n* = 20, average age = 20.89) was given acute exercise; Group 4 (*n* = 20, average age = 21.03) only watched a video without being given any acute exercise or music listening; Group 5 (*n* = 19, average age = 20.68) was given music listening after watching a video; Group 6 (*n* = 18, average age = 21.32) was given acute exercise after watching a video. Results: In the pre-test, we found that there was no significant difference in negative affect, positive affect, and cognitive performance among the groups (*p* > .05). The post-test indicated that the negative affect of college students who watched the video (20.16 ± 8.34) was higher than that of college students who did not watch the video (11.12 ± 3.29). Acute exercise and music listening improved the cognitive performance of college students with video-induced negative affect. Acute exercise improved the cognitive performance of college students with non-video-induced negative affect, while music listening did not. Conclusion: The acute decline in the cognitive performance of college students caused by video-induced negative affect can be ameliorated by means of acute exercise and music listening.

## 1. Introduction

In our daily life, many events and behaviors may induce negative affect, such as alcohol (Kasten et al. 2019), images (Gonzalo De La Casa et al. 2014), and social interaction (Kraus et al. 2020). Negative affect may further erode an individual’s cognitive achievement, leading to anger, panic, and disappointment (Di Santo et al. 2020). Long-term negative affect may lead to individual anxiety and depression (Barcaccia et al. 2022) and physical diseases, whereby negative emotions can aggravate the symptoms of chest pain (Stebenne et al. 2017). It has become a common laboratory practice to induce negative affect to observe its physical and psychological effects on individuals (Giannouli 2017). A study has shown that teens are likely to overeat when negative affect is induced using film materials (Czekalla et al. 2021). In previous studies, inducing negative affect in the subjects has aimed to effectively relieve the negative affect by providing a cure for the healthy individuals affected.

Studies have shown that exercise can relieve the negative affect of individuals. One study indicated that aerobic exercise can cause positive affect and relieve the individual’s anxiety (Abrantes et al. 2019). In the research on the effect of exercise on negative affect, most studies have found that frequency and intensity are the main factors in relieving negative affect (Fountaine et al. 2020; Garcia 2012). Some researchers have determined a significant negative correlation between exercise frequency and negative affect (Rodrigues et al. 2022). However, other researchers have put forward different views. Incremental exercise in a state of severe hypoxia can cause negative affect, indicating that exercise can also lead to negative affect (Keramidas et al. 2016). At present, the research on the effect of exercise on negative affect is controversial. There are also many studies citing music listening against negative affect, and more research results have been achieved. Groarke demonstrated how a speech preparation and arithmetic task were used as a treatment by playing music to intervene with participants with induced negative affect. It has been shown that music can significantly reduce the subjects’ anxiety and improve their attention (Groarke et al. 2020). Research has indicated that individuals who prefer intense music have lower levels of negativity. Through the analysis of music preferences, it is possible to indirectly predict the mental health level of individuals (Monteiro et al. 2021). Some studies have proved that patient-preferred live music can effectively relieve negative affect and reduce postoperative pain in cancer patients (Merry and Silverman 2021). Through the above research, both exercise and music have been shown to effectively relieve the negative affect of individuals, but which can better alleviate the negative affect of individuals requires further study.

Changes in cognition through exercise have been recognized by more and more researchers. Zhang used behavioral measures combined with event-related potentials (ERPs) to specifically investigate the impacts of negative affect evoked by implicit cues on conflict inhibition (flanker task), and whether acute exercise could mitigate these effects. The results demonstrated that negative flanker trials produced a larger N1 amplitude but smaller N200 amplitude than neutral trials; furthermore, acute exercise could mitigate emotional effects on N1 (Zhang et al. 2022). Roig pointed out that acute exercise improves memory in a time-dependent fashion by priming the molecular processes involved in the encoding and consolidation of newly acquired information. Therefore, acute exercise can improve human memory more (Roig et al. 2013). In Roig’s other review study, study showed that a single bout of cardiovascular excise can both improve memory (Roig et al. 2016). Roig’s research proves the beneficial effect of acute exercise on cognitive performance. It is not only acute exercise that has a beneficial effect on memory, but also chronic exercise. Through systematic review research, Loprinzi found that most studies show that acute and chronic exercise has a favorable effect on the memory function of healthy middle-aged adults, while exercise has better effect on the memory function of the nondepressed population (Loprinzi et al. 2018). However, there are mixed findings in this regard. The Montreal Cognitive Assessment Scale (MoCA) was used to evaluate the global cognition and mild cognitive impairment of the elderly. Five years of exercise was not found to affect cognition (Zotcheva et al. 2022). A meta-analysis pointed out that the effects of acute exercise on cognitive performance are generally small; however, larger effects are possible for particular cognitive outcomes and when specific exercise parameters are used (Chang et al. 2012). To study the effect of music on individual cognition, the application of music plus cognitive training was determined to improve cognition in the elderly more effectively than cognitive training alone (Castro Coelho et al. 2020) and music improved cognition not only in older adults but also in stroke patients (Haire et al. 2021). There remain contradictions in the research on the effect of music on improving cognition. A meta-analysis pointed out that patients with frontotemporal dementia can use music to activate the social cognition-related functional areas of the brain, proving that music plays a certain role in promoting cognition (Van’T Hooft et al. 2021). Another meta-analysis found that although both exercise and music can improve cognition in the elderly, the effect is small, indicating that in-depth research is needed to further prove the effect of music and exercise on improving cognition in the elderly (Yoo et al. 2022). There are few studies on exercise or music listening that have observed college students’ cognition after induced negative affect. Moreover, there is a lack of comparative studies on improving individual cognition using exercise and music. Therefore, further analysis of the effect of exercise and music on the improvement of individual cognition with induced negative affect is needed.

To explore the influence of acute exercise and music listening on the cognition of college students, we used video clips to temporarily induce negative affect and then measured their cognition four times. In this study, a video was used to induce negative affect in college students. Then, acute exercise and music listening were applied to college students. Finally, the Flanker task, Stroop task, 2-back task were used to evaluate the cognitive effects in college students. We hypothesized that negative affect would have an impact on their cognition; clips of movies could effectively induce negative affect in college students. In addition, we hypothesized that the improvement in individual cognition could only be maintained for a short time after a single acute exercise or music listening. This study tested whether acute exercise and music listening influenced the recuperation from an acute decline in cognitive performance induced by negative affect.

## 2. Materials and Methods

### 2.1. Participants

When recruiting subjects, we first used G*Power (3.1.9.7) under the genre of power analysis. According to the calculation method of the previous response size, .2 was a small effective size, and .5 was a medium effective size (McShane and Boeckenholt 2016). We selected an average effect size of .35 (alpha of .05; power of .8; number of measures, 4; number of groups, 6). After calculation, 72 subjects were sampled to meet the analysis requirements. The estimated attrition rate of this experiment was 20%, so at least 87 subjects needed to be recruited for the experiment.

According to the needs of this research, the subjects were openly recruited from Northeastern University. This study was approved by the Ethics Committee of Northeastern University (EC-2022B028). The included college students were all healthy adults who met the requirements. In this study, the criteria for included college students were as follows: (1) full-time college students; (2) all the subjects were right-handed; (3) voluntary participation in the experiment; and (4) filling in informed consent and reporting their physical and mental health.

In order to minimize the college students’ adverse effects from induced negative affect, the subjects who met any of the following criteria were excluded: (1) with mental illness; (2) with suicidal tendencies; (3) experienced major traumatic events; (4) receiving psychological or psychiatric treatment; (5) Self-rating Depression Scale (Zung 1965) reached a moderate depression level; and (6) Beak Self-rating Anxiety Scale (Beck et al. 2001) reached a moderate anxiety level. Participants were recruited beginning in March 2022. A total of 135 healthy college students were recruited to participate in this experiment. After excluding 19 subjects who could not participate for various reasons and dropped out halfway, 116 subjects were finally included in the statistical analysis (including 86 males and 30 females). All the participants were randomly divided into 6 groups. Participants were assigned following simple randomization procedures (computerized random numbers) to 1 of 6 groups: Group 1 (19), Group 2 (20), Group 3 (20), Group 4 (20), Group 5 (19), and Group 6 (18). One week after the end of the experiment, the subjects participating in the experiment were asked about their “emotional state after the end of the study” and “whether they were still negatively affected by the experimental video.” If any subjects had psychological problems caused by the experiment, they met with psychologists for psychological counselling.

### 2.2. Materials

#### 2.2.1. Materials That Induced Negative Affect

Films about relationship loss were selected to induce negative affect in the subjects. Relationship loss films were effective in inducing negativity affect in younger individuals (Ready and Santorelli 2016). The film montage used in the negative affect induction included clips from the films *Up* (an older man grieving the death of his wife), *Steel Magnolias* (a mother mourns at her daughter’s funeral), *Sophie’s Choice* (a Nazi soldier forcing a mother to choose one of her children to send to death), and *Pay It Forward* (a boy tries to intervene in a fight at school and was stabbed to death as a result). These films were shown to be effective in eliciting negative affect in younger viewers (Mather and Ready 2021). The films were edited and combined into a video. Each edited video segment had a title and lasted about 3 minutes; the total time was 12 minutes. Subjects were asked to watch the video alone in a dimly lit laboratory.

#### 2.2.2. Cognition Measurement

Inhibition control was measured by the flanker task (Eriksen and Eriksen 1974), the subjects’ response inhibition was measured by the Stroop task (Cai et al. 2013), and the 2-back task (Kirchner 1958) was used to measure the subjects’ working memory. Inhibitory control refers to the ability to suppress inappropriate responses (Chen et al. 2019); response inhibition is the capacity to suppress inappropriate actions and considered to be a fundamental executive function (Walther et al. 2010). The difference between the two is that inhibition control refers to inhibiting reaction speed, while response inhibition refers to controlling individual behavior. All programs were presented on E-prime 3.0.

The flanker task was used to measure inhibitory control. The experimental background was black. The stimuli were five white arrows displayed on the screen for 1000 ms. The subjects observed the direction of the middle arrow. The subject’s left index finger pressed the A key, and the right index finger pressed the L key. The subjects were asked to respond as soon as possible in the test types that were congruent (>>>>> or <<<<<) and incongruent (>><>> or <<><<). If there was congruence, they were to press the A key, and if there was an inconsistency, press the L key. The probability of congruent and incongruent was equal. Participants completed a total of 80 trials. Before the start of the formal experiment, there were 10 pre-trials. When the subjects completed the pre-trials, they were asked whether they were ready to start the formal experiment. If so, they officially entered the experiment. If they were not familiar with the experiment, they continued the 10 pre-trials. The experimental indicators used the incongruent reaction time and accuracy means of the subjects.

The Stroop task was used to measure response inhibition. The task stimuli consisted of words of different colors. There were four words in total: red, yellow, blue, and green. There were also four colors: red, yellow, blue, and green. The time that each word appeared was 1000 ms, and the subjects were required to judge the color of the word. For red, they were to press the R key; yellow, press the Y key; blue, press the B key; and green, press the G key. For the congruent, the font text and word colors matched; for the incongruent, the font text and word colors did not match. Congruent and incongruent accounted for 75% and 25% of the total number of trials, respectively. There were 60 trials in this test. Before the formal test began, there were 10 pre-trials. If the subjects were completely familiar with the experiment, the formal experiment would be conducted. Subjects needed to press the key within 2000 ms; otherwise, they were treated as wrong answers. This study used the reaction time of the incongruent trials as the main dependent variable for this task.

The 2-back task was used to measure working memory. First, the screen was equally divided into nine separated spaces. A 3 × 3 grid was presented to the participant with each stimulus involving a block appearing in one of the 9 spaces. The subjects needed to indicate whether the position of the square was congruent with the position before the previous two stimuli. If it was congruent, they pressed the A key, and if not, the L key. Each stimulus was presented for 500 ms, and the interval between two stimuli was 1500 ms. There was a total of 60 trials. Before the test, there were 10 per-trials. After they became proficient, the formal experiment would be entered for the test. Accuracy was the primary measure of working memory.

### 2.3. Acute Exercise and Music Listening 

The acute exercise group used a high-intensity exercise program. The exercise intensity was monitored in real time using the Borg RPE scale. During the exercise process, the subjects were first allowed to warm up for 5 min. During the warm-up, when the subjects reported the RPE reached 13, they could begin the acute exercise. During the exercise, the subjects undertook acute exercise. Treadmills (Model 8TRx, STAR TRAC, Irvine, CA, USA) were used for the acute exercise, and the real-time heart rate of the subjects was displayed on the treadmill. All exercise groups participated in the acute exercise. After the warm-up, the subjects ran on the treadmill. When their heart rate reached more than 80% of the maximum (maximum heart rate = 220 − age), they continued to exercise for 3 min. The treadmill speed was then adjusted, so that the heart rate of the subjects slowly dropped to about 50% of the maximum heart rate, and they continue to exercise for 3 min. A running cycle lasted for 6 minutes, and there were 3 running cycles in total.

The subjects in the music listening group listened to music. We asked the subjects to choose their favorite music and each subject used their mobile phone to store 6–8 pieces of their favorite music. The selected music had to be music that made them feel happy and relaxed. At the beginning, they played their favorite music while wearing headphones, and the music duration was 18 min. After the music listening groups finished listening to the music, there was an approximately 20 min break before returning to the laboratory to complete cognition tests. During these 20 min, the subjects had a quiet rest. In cognition tests, inhibition tests (flanker and Stroop) and working memory (2-back) were measured randomly.

### 2.4. Procedure

Group 1 was not given any form of acute exercise and music listening; Group 2 was given music listening; Group 3 was given acute exercise; Group 4 only watched the video without any acute exercise and music listening; Group 5 was given music listening after watching the video; Group 6 was given the acute exercise after watching the video.

**Figure 1:**
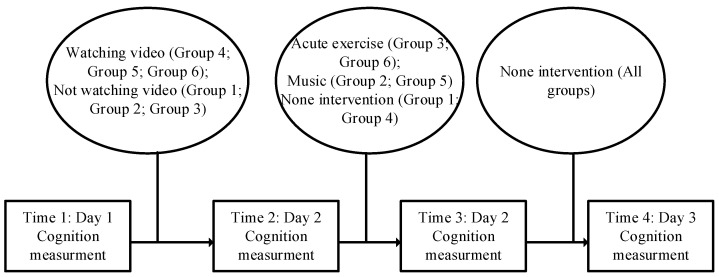
The experimental procedure.

Cognition was divided into four measurements over three days: Day 1: first measurement. Measurements were performed after recruitment and group assignment (time 1). Day 2: second and third measurements. The second measurement was performed after watching the experimental video, and the group that did not watch the film was also measured (time 2); the third measurement was performed after the music listening and acute exercise. The group without the music listening and acute exercise was also measured (time 3). Day 3: fourth measurement. All the subjects were remeasured in the morning of the second day after the acute exercise or music listening (time 4). Figure 1 contains a schematic overview of the experimental procedure. No adverse events were observed during the experiment.

### 2.5. Statistical Analysis

SPSS 26.0 was used for statistical analysis. First, all means and standard deviations (SD) were statistically analyzed using standardized statistical methods. The Shapiro–Wilk test was used for the normal distribution of the data, and Levene’s test was used for the homomorphic distribution. Partial Eta squared ($\eta_{p}^{2}$) was used to calculate effect sizes for significant main effects and interactions. Mauchly’s test of sphericity was used for the evaluation of the variance difference between different measurements. If the sphericity test was not met, the analysis was performed using Greenhouse–Geisser. A post hoc *t*-test was used to report estimated effect sizes. A mixed-effect analysis of variance 2 (induced negative affect: no/yes) × 3 (type: acute exercise, music listening, rest) × 4 (measurement time: time 1/time 2/time 3/time 4) model was used to count the subjects’ flanker, Stroop and 2-back tasks.

## 3. Results

### 3.1. Participant Characteristics

For the recruited subjects, a survey of basic information was conducted (Table 1). Gender (χ^2^(5) = .995, *p* > .05), age (*F*(5,110) = 1.314, *p* > .05), body height (*F*(5,110) = 1.243, *p* > .05), body weight (*F*(5,110) = 1.003, *p* > .05), BMI (*F*(5,110) = 1.215, *p* > .05), and resting heart rate (*F*(5,110) = 1.614, *p* > .05) were recorded. The results of the study showed that there was no significant difference in the basic conditions among the six groups, indicating that the subjects were grouped with good consistency.

### 3.2. Affective Measurement

**Figure 2:**
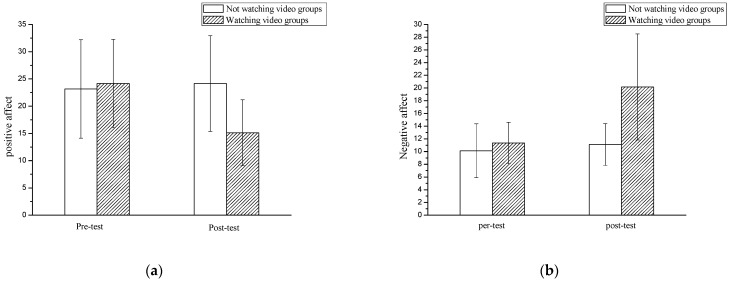
Comparison of positive affect and negative affect pre-test and post-test between the groups watching the video and the groups not watching the video ((**a**): positive affect score; (**b**): negative affect score).

Before the subjects watched the video, their affect was measured, and the Positive Affect and Negative Affect Scale (PANAS) (Watson et al. 1988) was used to measure the affect changes in the subjects before and after watching the video. PANAS consists of 20 questions, including 10 positive affect and 10 negative affect questions. Both positive affect and negative affect were calculated using Li Kete’s Five Scaling Method. Before and after watching the video, a paired sample *t*-test was conducted on the positive and negative affect of the group that did not watch the video and the group that watched the video. The results showed that there was no significant difference (*p* > .05) between the pre-test positive affect (23.15 ± 9.03) and the post-test positive affect (24.15 ± 8.79) of the groups that did not watch the video; there was no significant difference (*p* > .05) between the pre-test negative affect (10.13 ± 4.23) and the post-test negative affect (11.12 ± 3.29). There was a significant difference (*p* < .05) between the pre-test positive affect (24.15 ± 8.10) and the post-test positive affect (15.12 ± 6.05) of the groups who watched the video. The pre-test negative affect (11.34 ± 3.26) and the post-test negative affect (20.16 ± 8.34) were significantly different (*p* < .05), indicating that the video can effectively induce affect changes in the subjects. Studies have suggested that personality may have an impact on negative affect (Hisler et al. 2020; Miller et al. 2009). In our study, after watching the video, that group’s negative affect was higher than the group that did not watch the video, which demonstrated that our grouping was reasonable. The descriptive statistical results are shown in Figure 2 and Table A1.

### 3.3. Cognition

#### 3.3.1. Inhibitory Control—Flanker Task

**Figure 3:**
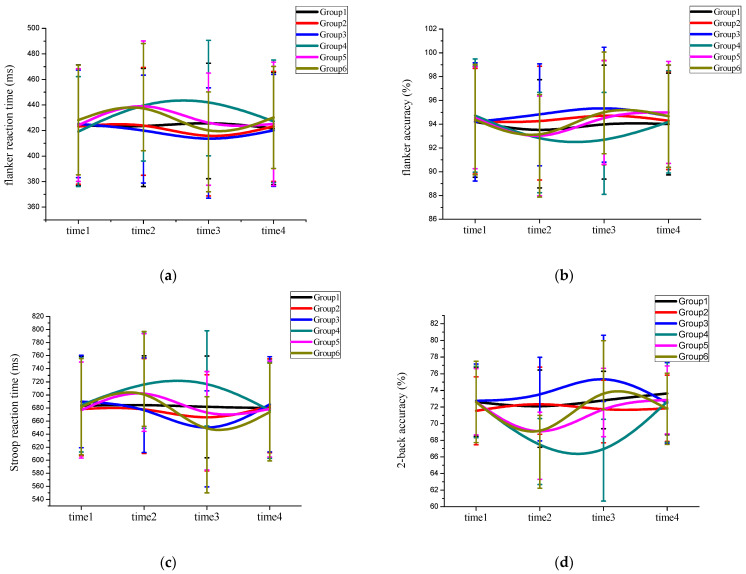
Cognition changes across the four measurements ((**a**): flanker task reaction time; (**b**): flanker task accuracy; (**c**): Stroop task reaction time; (**d**): 2-back task accuracy. time1: End of recruitment measurement; time2: Measurement after watching video or not watching video; time3: Day 2 measurement after (no) acute exercise or music listening; time4: Without any acute exercise and music listening measurement. Group 1 was not given any form of acute exercise and music; Group 2 was given music listening; Group 3 was given acute exercise; Group 4 watched the video without any acute exercise and music listening; Group 5 was given music listening after watching the video; Group 6 was given the acute exercise after watching the video).

Reaction time: The omnibus analysis for flanker reaction time revealed a main effect of time, *F*(3,113) = 48.13, *p* < .001, $\eta_{p}^{2}=.56$. After the post hoc test, it was found that the reaction time of the third measurement (420.15 ± 38.93 ms) was significantly shorter than that of the first measurement (448.15 ± 45.64 ms) and of the fourth measurement (450.12 ± 47.87 ms). Both were shorter than the second measured reaction time (467.18 ± 49.35 ms). The main effect of induced negative affect was significant, *F*(3,112) = 146.14, *p* < .001, $\eta_{p}^{2}=.79$. The post hoc test found that the reaction time of the subjects without video-induced negative affect was lower than the subjects who had video-induced negative affect and there were significant differences. The main effect of the acute exercise and music listening type was significant, *F*(3,111) = 87.70, *p* < .001, $\eta_{p}^{2}=.69$. The post hoc test found that the reaction time of the acute exercise groups and music listening groups were not significant, but both were lower than those of the groups with no acute exercise or music listening. The interaction between measurement time and induced negative affect was significant, *F*(3,112) = 142.60, *p* < .001, $\eta_{p}^{2}=.79$. Further simple effect analysis showed that after induced negative affect, the reaction time of the induced negative affect groups (447.13 ± 40.25 ms) were significantly higher than those of the non-induced negative affect groups (428.19 ± 39.19 ms), *F*(1,114) = 485.12, *p* < .001, $\eta_{p}^{2}=.81$. The interaction between measurement time and the acute exercise and music listening type was significant, *F*(6,224) = 20.10, *p* < .001, $\eta_{p}^{2}=.35$. Further simple effect analysis showed that after acute exercise and music listening, the reaction times for the acute exercise groups (418.36 ± 38.90 ms) were lower than those of the music listening groups (423.19 ± 40.13 ms), and they were significantly lower than the reaction times of the groups with no acute exercise or music listening (439.19 ± 48.49 ms), *F* (2,113) = 79.63, *p* < .001, $\eta_{p}^{2}=.59$. The effect size ($\eta_{p}^{2}=.35$) of the acute exercise and music listening type was lower than the effect size ($\eta_{p}^{2}=.79$) of induced negative affect (Figure 3a and Table A2).

Accuracy: The omnibus analysis for flanker accuracy revealed a main effect of time, *F*(3,113) = 37.63, *p* < .001, $\eta_{p}^{2}=.50$. After the post hoc test, it was found that the accuracy of the third measurement (94.37 ± 6.23), the accuracy of the first measurement (94.20 ± 4.15), and the accuracy of the fourth measurement (94.21 ± 4.22) were higher than that of the second measurement (93.35 ± 5.13). The main effect of induced negative affect was significant, *F*(3,112) = 136.74, *p* < .001, $\eta_{p}^{2}=.54$. The post hoc test found that the accuracy of the subjects with video-induced negative affect was lower than the subjects who were without video-induced negative affect and there were significant differences. The main effect of the acute exercise and music listening type was significant, *F*(3,111) = 46.08, *p* < .001, $\eta_{p}^{2}=.55$. The post hoc test found that the accuracy of the acute exercise groups was higher than that of the music listening groups and the groups with no acute exercise or music listening. The interaction between measurement time and induced negative affect was significant, *F*(3,112) = 283.85, *p* < .001, $\eta_{p}^{2}=.88$. Further simple effect analysis showed that after induced negative affect, the accuracy of the induced negative affect groups (92.21 ± 3.16) was significantly lower than that of the non-induced negative affect groups (94.25 ± 4.33), *F*(1,114) = 1117.17, *p* < .001, $\eta_{p}^{2}=.91$. The interaction between measurement time and the acute exercise and music listening type was significant, *F*(6,224) = 17.28, *p* < .001, $\eta_{p}^{2}=.31$. Further simple effect analysis showed that after the acute exercise and music listening, the accuracy of the acute exercise groups (95.52 ± 4.29) was higher than that of the music listening groups (94.24 ± 5.13), and it was significantly higher than the accuracy of the groups with no acute exercise or music listening (93.32 ± 4.86), *F*(2,113) = 81.29, *p* < .001, $\eta_{p}^{2}=.59$. The effect size ($\eta_{p}^{2}=.31$) of the acute exercise and music listening type was lower than the effect size ($\eta_{p}^{2}=.88$) of induced negative affect (Figure 3b and Table A3).

#### 3.3.2. Response Inhibition—Stroop Task

Reaction time: The omnibus analysis for Stroop reaction time revealed a main effect of time, *F*(3,113) = 51.98, *p* < .001, $\eta_{p}^{2}=.58$. After the post hoc test, it was found that the reaction time of the third measurement (664.66 ± 72.35 ms) was significantly shorter than those of the first measurement (682.585 ± 69.39 ms) and the fourth measurement (685.34 ± 76.32 ms), and both shorter than the second measured reaction time (701.34 ± 70.49 ms). The main effect of induced negative affect was significant, *F*(3,112) = 181.88, *p* < .001, $\eta_{p}^{2}=.83$. The post hoc test found that the reaction time of the subjects without video-induced negative affect was lower than the subjects who had video-induced negative affect and there were significant differences. The main effect of the acute exercise and music listening type was significant, *F*(3,111) = 122.12, *p* < .001, $\eta_{p}^{2}=.76$. After the post hoc test, the reaction time of the acute exercise group was shorter than the music listening groups, and both were lower than those of the groups with no acute exercise or music listening. The interaction between measurement time and induced negative affect was significant, *F*(3,112) = 167.90, *p* < .001, $\eta_{p}^{2}=.81$. Further simple effect analysis showed that after induced negative affect, the reaction time of the induced negative affect groups (720.13 ± 78.03 ms) were significantly higher than those of the non-induced negative affect groups (682.47 ± 70.47 ms), *F*(1,114) = 601.37, *p* < .001, $\eta_{p}^{2}=.84$. The interaction between measurement time and the acute exercise and music listening type was significant, *F*(6,224) = 23.60, *p* < .001, $\eta_{p}^{2}=.38$. Further simple effect analysis showed that after acute exercise and music listening, the reaction times of the acute exercise groups (632.74 ± 73.28 ms) were lower than those of the music listening groups (658.89 ± 78.92 ms), and they were significantly lower than the reaction times of the groups with no acute exercise or music listening (701.23 ± 67.89 ms), *F*(2,113) = 159.13, *p* < .001, $\eta_{p}^{2}=.73$. The effect size ($\eta_{p}^{2}=.38$) of the acute exercise and music listening type was lower than the effect size ($\eta_{p}^{2}=.81$) of induced negative affect (Figure 3c and Table A4).

#### 3.3.3. Working Memory—2-Back Task

Accuracy: The omnibus analysis for working memory accuracy revealed a main effect of time, *F*(3,113) = 41.85, *p* < .001, $\eta_{p}^{2}=.52$. After the post hoc test, it was found that the accuracy of the third measurement (74.92 ± 5.18) was higher than that of the first measurement (72.63 ± 3.11) and the fourth measurement (72.97 ± 4.02), but both were higher than the second measurement (70.63 ± 3.51). The main effect of induced negative affect was significant, *F*(3,112) = 96.92, *p* < .001, $\eta_{p}^{2}=.72$. The post hoc test found that the accuracy of the subjects with video-induced negative affect was lower than subjects who had no video-induced negative affect and there were significant differences. The main effect of the acute exercise and music listening type was significant, *F*(3,111) = 75.28, *p* < .001, $\eta_{p}^{2}=.67$. The post hoc test found that the accuracy of the acute exercise groups was higher than the music listening groups and groups with no acute exercise or music listening. The interaction between measurement time and induced negative affect was significant, *F*(3,112) = 62.68, *p* < .001, $\eta_{p}^{2}=.62$. Further simple effect analysis showed that after induced negative affect, the accuracy of the induced negative affect groups (72.83 ± 3.89) was significantly lower than that of the non-induced negative affect groups (77.08 ± 4.57), *F*(1,114) = 266.37, *p* < .001, $\eta_{p}^{2}=.70$. The interaction between measurement time and the acute exercise and music listening type was significant, *F*(6,224) = 75.23, *p* < .001, $\eta_{p}^{2}=.36$. Further simple effect analysis showed that after the acute exercise and music listening, the accuracy of the acute exercise groups (73.18 ± 4.96) was higher than that of the music listening groups (72.76 ± 5.13), and it was significantly higher than the accuracy of the groups with no acute exercise or music listening (66.41 ± 4.15), *F*(2,113) = 159.17, *p* < .001, $\eta_{p}^{2}=.73$. The effect size ($\eta_{p}^{2}=.36$) of the acute exercise and music listening type was lower than the effect size ($\eta_{p}^{2}=.62$) of induced negative affect (Figure 3d and Table A5).

## 4. Discussion

This study not only proved that a film about relationship loss can effectively induce affect changes in healthy individuals, but also concluded that it can induce cognition changes through cognitive testing. After analysis, both acute exercise and music listening improved the subjects’ cognitive levels, which was consistent with our hypothesis. In cognitive tests, the effect size of induced negative affect was higher than that of the acute exercise and music listening type. Lyons point out that negative affect mediated the association between all post-traumatic cognition types (Lyons et al. 2020). This study has shown that negative affect had a greater impact on cognitive functions, which is consistent with Lyons’ research. However, according to the results of the study, there was no difference between the last cognition measurement and the first cognition measurement of the subjects after induced negative affect, indicating that its effect on cognition cannot last for three days. Moreover, the last cognitive test of the subjects who had undergone acute exercise and music listening was no different from the first cognitive test, indicating that a single acute exercise and music listening activity could not maintain long-term improved cognition. After analyzing the measurement results of all groups, we found that acute exercise and music listening did not improve cognition compared to the baseline, but helped it to bounce back from the negative effect of the videos.

A unique feature of the design of this study was that it first induced negative affect in the subjects, and we then used acute exercise and music listening to compare the effects. This study demonstrated that while acute exercise and music listening can improve cognition, the effect size is lower compared to video-induced negative affect. Another study showed that individuals with low positive affect experience greater cognitive and positive affect improvements following acute aerobic exercise at a self-selected intensity (M. N. Johnson et al. 2022). This study indicated that in the Stroop task, the reaction time of subjects induced by negative affect increased significantly. In the flanker task, the subjects with induced negative affect not only increased their reaction time, but also decreased in accuracy. In the 2-back task, this study found that the accuracy of subjects induced by negative affect also decreased. Therefore, it suggested that the impact of negative affect on cognition is comprehensive, leading not only to the decline in inhibition, but also to the decline of working memory.

Regarding the results of acute exercise and music listening on cognition, acute exercise effectively improved the Stroop task and 2-back task of the subjects whether they had video-induced negative affect or not. On the one hand, acute exercise effectively improved the cognition of subjects who had video-induced negative affect. On the other hand, music listening also effectively reduced the Stroop and flanker task reaction time, and improved the accuracy of the Stroop task. However, the music listening had no effect on the accuracy of the flanker task and 2-back task in subjects without induced negative affect, and the difference in reaction time between the Stroop task and the flanker task was not significant. Music listening caused a certain improvement in the accuracy of the flanker task and 2-back task in subjects who had induced negative affect and had a significant effect on the reaction times of the Stroop task and flanker task. Therefore, music listening improved the cognitive decline of college students caused by inducing negative affect. Acute exercise improved cognition in college students with or without video-induced negative affect. Comparing acute exercise and music listening, a single session of acute exercise seemed to improve the subjects’ cognition more than a single session of music listening.

The main explanation for the improvement in individual cognition caused by acute exercise is that acute exercise can improve individual physiology; in particular, it will lead to an increase in brain derived neurotrophic factor (BNDF), which can promote the growth and development of the central nervous system and peripheral neurons, and maintain the normal function of nervous system (T. K. Johnson et al. 2020). Acute exercise can effectively improve maximum oxygen uptake, thus effectively improving the level of individual physiology. Some studies have proposed an enteroception model for the acute exercise–cognition interaction; the effect of exercise on cognition may be related to the release of catecholamines (McMorris 2021). Catecholamine is a kind of nerve substance containing catechol and amine groups. Catecholamine can act on sympathetic nerves, and then effect cognitive function. Therefore, it is reasonable to believe that acute exercise can promote changes in some physiological indexes in college students. A study has shown that when magnetic resonance imaging (MRI) is used to measure working memory, the mechanism of exercise-induced improvement in working memory may be associated with lower grey matter blood flow (GMBF). The main manifestation was that after 30 min of aerobic exercise, GMBF decreased, and GMBF was negatively correlated with working memory accuracy (Olivo et al. 2021). This indicates that acute exercise can lead to a change in blood flow, and then affect the working memory of individuals. Some research has suggested that acute exercise-induced lowering of negative affect may be mediated by the bilateral amygdala in relation to the functional connectivity of cognitive control and limbic networks, because exercise groups exhibited increased functional connectivity between the right amygdala and right orbital frontal cortex (Ge et al. 2021), suggesting that acute exercise can effectively improve individual cognition. The influence of music listening on cognition can be explained as follows. The mechanism whereby music listening improves cognition is mainly that music listening causes declines in the fronto-temporal functional connectivity and radial diffusivity of dorsal white matter, perhaps caused by a reduced need of top-down control as the task becomes more familiar (Fischer et al. 2021). This shows that music listening has a strong plasticity for the nervous system, and then affects individual cognition. Above research demonstrated that the effect of acute exercise and music listening on brain function is a major cause of cognition changes.

Although we did not conduct long-term follow-up trials, the results of the single exercise and music treatments indicated cognitive improvement for college students. The result also showed that while a single session of the acute exercise and music listening can improve college students’ cognition, the improvement time remains short. In view of the practical application value of this research, in ordinary life, individuals are always unavoidably disturbed by negative affect caused by work pressure and setbacks. Acute exercise and music listening can be used as a temporary relief therapy for individuals who normally experience negative affect. If individuals suffering from negative affect can exercise, acute exercise can be used to relieve negative affect. If exercise is not possible, music listening can be used to relieve negative affect. In short, acute exercise can effectively improve the individual’s cognition.

Although the effects of both acute exercise and music listening on the cognition of college students with induced negative affect were proved, acute exercise is more effective. This study also has certain limitations. First, the self-evaluation of affect is biased, and individuals may see the negative-affect-inducing material differently, producing different results. Second, this study only conducted experiments on college students and lacked comparison experiments with other age groups. Therefore, it is not known whether acute exercise and music listening have the same effect on other age groups. Third, we only examined the inhibition and working memory aspects of cognition and did not consider the results of other aspects such as reasoning and flexibility and so on. Fourth, because the average level of cognition of college students was investigated in this study, personality and intelligence were not considered in this study, and the difference between personality and intelligence may have a certain impact on the results. In future research, we can conduct multi-faceted investigations. 

## 5. Conclusions

Overall, the results of this paper indicate that video-induced negative affect reduces the cognitive level of college students. Acute exercise and music listening improve the cognitive level of college students with video-induced negative affect. The acute exercise effect is better in this regard. Moreover, acute exercise improves the cognitive level of non-video-induced college students, while music listening does not. Therefore, combined with previous studies, the study finds that acute exercise is an effective means to improve the cognition of college students.

## Figures and Tables

**Table 1:** Participant characteristics (M ± SD).

	Group1	Group2	Group3	Group4	Group5	Group6	*p*-Value
M/F	14/5	15/5	14/6	16/4	13/6	14/4	.963
Age (year)	20.15 ± 2.97	21.33 ± 2.76	20.89 ± 2.77	21.03 ± 2.19	20.68 ± 2.68	21.32 ± 2.55	.205
Body height (cm)	173.14 ± 12.52	172.13 ± 10.39	172.87 ± 12.11	173.15 ± 11.36	171.67 ± 10.53	173.06 ± 12.68	.211
Body weight (kg)	61.23 ± 10.33	62.75 ± 11.21	61.89 ± 9.93	63.05 ± 9.76	61.07 ± 10.68	62.04 ± 11.21	.101
BMI (kg/m^2^)	20.53 ± 2.85	21.12 ± 2.86	20.96 ± 2.68	21.17 ± 2.77	20.92 ± 2.75	20.57 ± 2.31	.224
Resting heart rate (times/min)	72.15 ± 11.36	74.13 ± 10.58	73.92 ± 12.11	72.18 ± 13.15	72.48 ± 12.92	73.94 ± 13.28	.315

Note: M: mean; SD: standard deviations; BMI: Body Mass Index.

## Data Availability

The data presented in this study are available on request from the corresponding author. The data are not publicly available due to privacy reasons.

## Appendix A

**Table A1:** Comparison of positive affect and negative affect pre-test and post-test between the groups watching the video and the groups not watching the video.

Group		Not Watching the Video		Watching the Video			
		Mean	SD	Mean	SD	*p*-Value	Cohen’s d
Positive affect	Pre-test	23.15	9.03	24.15	8.10	.613	.116
	Post-test	24.15	8.79	15.12	6.05	.000	1.196
Negative affect	Pre-test	10.13	4.23	11.34	3.26	.578	.320
	Post-test	11.12	3.29	20.16	8.34	.000	1.426

Note: M: mean; SD: standard deviations.

**Table A2:** Flanker reaction time (ms).

	Group1		Group2		Group3		Group4		Group5		Group6	
	Mean	SD	Mean	SD	Mean	SD	Mean	SD	Mean	SD	Mean	SD
time1	424.16	47.24	423.17	45.18	425.19	42.1	426.18	43.09	424.19	44.01	428.18	42.9
time2	422.39	46.29	423.19	42.19	425.13	42.19	443.12	47	447.18	42.96	446.17	41.98
time3	427.49	45.18	411.14	42.38	410.21	43.19	445.38	45.24	421.14	43.89	411.13	39.07
time4	421.53	43.91	423.19	43.1	425.13	43.83	423.19	47.92	425.18	48.32	422.19	39.94

Note: M: mean; SD: standard deviations.

**Table A3:** Flanker accuracy (%).

	Group1		Group2		Group3		Group4		Group5		Group6	
	Mean	SD	Mean	SD	Mean	SD	Mean	SD	Mean	SD	Mean	SD
time1	94.21	4.67	94.25	4.44	94.19	4.97	94.72	4.76	94.53	4.28	94.38	4.65
time2	93.19	4.55	94.09	4.78	94.78	4.29	92.46	4.22	92.17	4.19	92.18	4.31
time3	94.17	4.79	94.98	4.39	95.64	4.83	92.38	4.29	94.98	4.34	95.79	4.28
time4	94.01	4.28	94.29	4.11	94.67	4.29	94.19	4.3	94.99	4.29	94.67	4.31

Note: M: mean; SD: standard deviations.

**Table A4:** Stroop reaction time (ms).

	Group1		Group2		Group3		Group4		Group5		Group6	
	Mean	SD	Mean	SD	Mean	SD	Mean	SD	Mean	SD	Mean	SD
time1	683.42	75.73	678.53	71.72	689.90	70.81	684.52	71.93	676.81	73.55	681.96	73.63
time2	685.21	74.56	682.97	72.52	684.34	72.38	721.62	72.92	718.95	74.72	724.51	72.62
time3	681.54	77.94	657.32	73.84	632.72	73.55	725.32	72.81	660.46	75.37	623.61	73.72
time4	679.96	74.92	683.24	71.96	685.63	72.81	676.42	73.66	678.56	73.51	673.72	74.83

Note: M: mean; SD: standard deviations.

**Table A5:** 2-back accuracy (%).

	Group1		Group2		Group3		Group4		Group5		Group6	
	Mean	SD	Mean	SD	Mean	SD	Mean	SD	Mean	SD	Mean	SD
time1	72.63	4.16	71.55	4.09	72.75	4.41	72.6	4.29	72.63	3.99	72.61	4.89
time2	71.79	4.65	72.75	4.03	72.95	5.03	66.65	3.97	67.34	4.04	66.61	4.39
time3	72.84	3.45	71.46	3.76	76.65	3.97	65.61	4.92	72.53	4.11	75.83	4.14
time4	73.63	4.96	71.85	3.99	72.5	4.83	72.65	4.89	72.85	4.08	71.78	4.29

Note: M: mean; SD: standard deviations.
